# Post-stroke limitations in daily activities: experience from a tertiary care hospital in Ethiopia

**DOI:** 10.1186/s12883-023-03419-9

**Published:** 2023-10-09

**Authors:** Salhadin Mohammed, Jemal Haidar, Biniyam A. Ayele, Yared Mamushet Yifru

**Affiliations:** 1 Internal Medicine Department, Neurology Unit, School of Medicine, College of Health Sciences, Wollo University, Dese, Ethiopia; 2 School of Public Health, College of Health Sciences, Addis Ababa University, Addis Ababa, Ethiopia; 3 Neurology Department, School of Medicine, College of Health Sciences, Addis Ababa University, Addis Ababa, Ethiopia

**Keywords:** Stroke, Activity limitations, Facilities, Disabling factors, Ethiopia

## Abstract

**Background:**

The disability of stroke patients remains an important global health problem; yet information on the extent of restriction from basic and instrumental activities of daily living is limited, particularly in lower-and middle-income (LMIC) countries. Therefore, we examined the issue under the caption, since it is the first step in planning several rehabilitation services.

**Method:**

A facility-based cross-sectional study was done to assess the magnitude and predictors of post-stroke limitations in basic activities of daily living (BADL) using the Barthel Index (BI) scale and instrumental activities of daily living (IADL) using the Frenchay Activities Index (FAI) scale among patients who visited Tikur Anbessa Specialized Hospital in Addis Ababa, Ethiopia, Neurology Clinic from April-October, 2022. All patients having a diagnosis of stroke for more than six months duration were enrolled. Descriptive and inferential statistical analyses were done, and measures of estimated crude and adjusted odds ratio with 95% CI were constructed and a p-value less than 0.05 was considered statistically significant. The results are presented in figures and tables.

**Results:**

A total of 150 stroke patients were enrolled in the present study. The mean age of participants was 53 (14.9) years with slight male preponderance (51.3%). Ischemic stroke was present in 106 (70.7%) of them, while 44 (29.3%) had hemorrhagic stroke. Of this, 57 (38%) and 115 (79.3%) of them had limitations in basic and instrumental ADL, respectively. Comorbid cardiac disease (AOR = 6.9; 95%CI = 1.3–37.5) and regular substance use (AOR = 11.1; 95%CI = 1.1–115) were associated with limitations in BADL, while an increase in age (AOR = 1.1; 95%CI = 1.04–1.15) was associated with severe limitations in BADL. Initial stroke severity (AOR = 7.3; 95%CI = 1.2–44.7) was associated with limitations in IADL, whereas depression (AOR = 5.1; 95%CI = 1.1–23.2) was identified as a predictor of severe limitation in IADL.

**Conclusion:**

Limitation in activities of daily living (ADL) after stroke is common among Ethiopian patients. Therefore, screening for post-stroke limitations in daily activities is essential for further management and rehabilitative plans.

**Supplementary Information:**

The online version contains supplementary material available at 10.1186/s12883-023-03419-9.

## Background

Stroke is regarded as one of the most devastating neurologic disorders affecting around 14 million people worldwide each year [1]. The global burden is shared nationally, with over 52,500 incident cases reported in Ethiopia in the year 2016 [2]. World Stroke Organization (WSO) estimates that over 116 million years of healthy life are lost each year due to stroke-related disability [1]. In one UK study, stroke was associated with the highest odds of severe overall disability, affecting more domains of disability when compared to other disabling conditions like cardiac, mental, and pulmonary diseases [3].

ADL is a concept introduced in 1950 by Sydney Katz. The term collectively describes fundamental skills required to independently care for oneself [4]. It is classified into two types namely basic activities of daily living (BADLs) and instrumental activities of daily living (IADLs). BADL refers to those skills required to manage one’s basic physical needs [4], while IADLs refer to more complex activities related to the ability to live independently in the community [4]. IADLs tend to capture the patient’s ability to live independently in the home and assess a variety of activities like cooking, home management, and recreation. Disability is a long-term physical, mental, intellectual, or sensory impairment that hinders full and effective participation in society on an equal basis with others [5]. Both types of ADL are considered domains of disability and are subject to be affected in individuals with stroke-related motor and cognitive dysfunction.

Previous studies attempted to assess the post-stroke health-related quality of life (HRQOL) [6], in Ethiopia and documented unmet supportive care needs [7] with marked psychological consequences of stroke [8]. On the other hand, anecdotal data are suggestive of high levels of disability among stroke patients [6, 7]; though data on the level of disability, with limitations in basic and instrumental activities of daily living is scanty among stroke survivors in low and middle-income countries (LMIC) including Ethiopia. In view of this, we examined the extent of limitations in basic and instrumental activities of daily living among stroke patients. Moreover, we tried to identify the contributory factors for both basic and instrumental activity limitations and bridge the existing gap of knowledge for future program initiatives.

## Methods

### Study setting, population, and period

The study was conducted in Tikur Anbessa Specialized Hospital (TASH) from April to October 2022. TASH is located in the capital city of Ethiopia and is the largest government-owned hospital serving as a teaching hospital of Addis Ababa University and a major referral center for the entire country [7]. The hospital provides various services among which the neurology department is considered one of the milestone activities initiated in the country and renders various neurology-related activities such as stroke management. The stroke unit which was recently established operates 24 h, all days of the week and there is a dedicated stroke clinic to treat stroke survivors.

### Overview of Acute Stroke Admission and Care at Tikur Anbessa Specialized Hospital (TASH), since the establishment of a Standard of Care Management for Stroke (from November 2021-October 2022)

A total of 141 patients with acute stroke were admitted during an approximate period of one year (from November 2021 to October 2022). Of this, 75 (53.2%) were men. The mean age of admitted patients was 52 years (SD 17.8). Ischemic stroke was diagnosed in 89 (63.1%) of the patients, while the rest 52 (36.9%) had a hemorrhagic stroke. At arrival, the severity of the stroke was assessed using the NIHSS by trained neurology attendings or residents, and results were documented on patients’ charts. As a standard of care, all patients with suspected stroke receive an NIHSS Score at arrival, and this has been fully implemented at our hospital since November 2021. The median (IQR) NIHSS Score of admitted stroke patients during this period was 11 [6, 9]. A total of 124 (87.9%) had a moderate to severe stroke as per the NIHSS, while only 17 (12.1%) had a mild stroke. The inflated number of patients with moderate to severe stroke highlights the fact that TASH offers tertiary-level care for patients across the country.

### Study design and participants

A health facility-based cross-sectional study was employed to assess the magnitude and predictors of limitations in activities of daily living among stroke patients that had a follow-up for over six months. The initial follow-up need not be in our specialized hospital and included patients that were referred from other hospitals or transfer-ins. The main eligibility criteria for enrollment of patients were an age of > 18 years and stroke survivors with a stroke duration of more than 6 months. Patients with a stroke duration of less than 6 months were excluded from the study because the tool used to assess IADL is validated for a duration of stroke of 6 months or more [9–11].

The sample size was determined using a single population proportion formula with the assumptions of a 95% level of confidence and a 5% margin of error. Since we could not find any previous studies conducted in Ethiopia to determine limitations in ADL among adult stroke survivors, a proportion value of 50% was used. Assuming a proportion value of 50% would give us the largest sample size to detect a statistically significant difference. Based on these assumptions, a sample size of 384 was obtained. Using a correction formula for a finite population of approximately 200, with a 10% non-response rate, the final sample size was approximated to 150.

A consecutive sampling technique was used to select the participants till the sample size was reached.

### Data collection and test procedure

A pretested structured interviewer-administered questionnaire, which contains the socio-demographic, clinical (including depression status), and stroke-related factors was used to collect the data.

BADL was assessed using the Barthel Index (BI), which measures ten basic aspects of self-care and physical dependency (12–13). The 15-item Frenchay Activities Index (FAI), specifically developed for use with stroke patients [9–11], was used in the study to assess IADL.

All data were collected by trained clinicians by relevant guidelines and regulations. Those clients who volunteered to participate in the study were interviewed accordingly.

### Data entry, processing, and analysis

Data were entered and analyzed using SPSS version 25 software. Descriptive statistics for the BI and FAI are presented in a table for each item as a proportion. Models of the predictors of activity limitation (both basic (BI < 95) and instrumental (FAI < 30) were developed using binary logistic regression. Bivariate analysis was done to check the existence of crude association and to select candidate variables; those variables which were a-priori clinically important and having (p < 0.25) were included in the final multivariable model. The summary measures of estimated adjusted odd’s ratio (AOR) with 95% CI were constructed and a p-value of less than 0.05 was used to declare statistical significance.

### Definitions

#### Post-stroke Limitations in BADL (assessed by the Barthel Index (BI))

A score of 100 is considered normal, and lower scores indicate increasing disability as per the BI; a BI < 95 corresponds to assisted independence (limitations in BADL) [14], while a BI ≤ 60 corresponds to severe dependency (severe limitation in BADL) [15].

**Post-stroke Limitations in IADL (assessed by the Frenchay Activity Index (FAI))**: FAI score ranges: from 0(severely restricted) to 45(very active or unrestricted) and can be classified as Scores 31–45(Unrestricted/No limitations in IADLs); FAI score of ≤ 30 defines restriction/limitations in IADL. Scores ranging from 0 − 15 correspond to severe restriction.

#### Initial stroke severity

National Institute of Health Stroke Scale (NIHSS); defined cut points for mild, moderate, and severe stroke are not well established, but cut-points of NIHSS score as stated may be reasonable; <5 for mild, ≥ 5 for moderate to severe stroke.

**Depression**: Based on the PHQ-9 validated depression screening tool in general practice settings; a score of < 10: No or minimal Depression, while a Score of 10 or above refers to the presence of Depression [8].

**Cognitive Impairment**: Mini-mental state exam (MMSE) tool was used to screen for cognitive impairment; 24–30 points: No Cognitive Impairment, ≤ 23: Has Cognitive Impairment.

#### Modified Rankin Scale (mRS)

Score of.

- 0- No symptoms at all,
- 1- No significant disability despite symptoms; able to carry out all usual duties and activities,
- 2- Slight disability; unable to carry out all previous activities, but able to look after own affairs without assistance,
- 3- Moderate disability; requiring some help, but able to walk without assistance,
- 4- Moderately severe disability, unable to walk without assistance and unable to attend to own bodily needs without assistance,
- 5- Severe disability; bedridden, incontinent, and requiring constant nursing care and attention,
- 6- Dead.

#### Regular substance use

refers to as ever using a substance daily or nearly daily for 3 months or more [16]. The substances identified in our study are cigarette, alcohol, and khat (*Catha edulis*) use.

## Results

A total of one hundred and fifty stroke patients on follow-up were included with a response rate of 100%. The mean age was 53(14.9) years and 51.3% were males. 73.3% were married and 79.4% had formal education. 90.0% were urban residents. 56.7% of the patients had no sustainable income and 16.7% used substances regularly (Table 1).

**Table 1:** Baseline Socio-demographic characteristics of stroke patients attending TASH, Apr-Oct, 2022

Socio-demographic Characteristics		Frequency	%
Age (in Years); Mean (± SD)		53 (± 14.9)	
Gender	Female	73	48.7
	Male	77	51.3
Marital Status	Married	110	73.3
	Never Married	21	14.0
	Divorced	8	5.3
	Widowed	11	7.3
Residence	Urban	135	90.0
	Rural	15	10.0
Education	No Formal Education	22	14.7
	Reading and Writing	9	6.0
	Primary	25	16.7
	Secondary	48	32.0
	More than Secondary	46	30.7
Employment	Employed	35	23.3
	Employed (on paid leave)	6	4.0
	Retired	24	16.0
	Unemployed	55	36.7
	Housewife	28	18.7
	Student	2	1.3
Has sustainable Income Status	Yes	65	43.3
	No	85	56.7
Regular Substance Use *	Yes	25	16.7
	No	125	83.3

*identified substances in the study were alcohol, nicotine, and khat (*Catha edulis*)

### Clinical characteristics

As shown in Table 2, Ischemic stroke was diagnosed among 106 (70.7%) stroke survivors. Concerning the time from the last stroke attack, 97 (64.67%) of them had their last stroke attack within the last one year. A history of stroke recurrence was reported among 14 (9.3%) of them. Hypertension and cardiac diseases were the most common medical comorbidities reported among 105 (70%) and 43 (28.7%), respectively. Sixty-five (43.3%) of them screened positive for depressive symptoms (PHQ-9 score of ≥ 10), with the median (IQR) PHQ-9 score being 6 [2–8, 12, 17]. The median (IQR) NIHSS, mRS, and MMSE scores were 10 [5–8, 12, 13, 17, 18], 2 [1–3], and 26 [19–24], respectively; while the median (IQR) duration since the last stroke was 9.5 months (6–36 months).

**Table 2:** Clinical Characteristics of Stroke Patients attending TASH, April-Oct, 2022

Clinical Characteristics	Categories	Number	%
Time Since Last Stroke	≤ 12 months	96	64.0
	> 12 months	54	36.0
Age at Onset	≤ 50 years	74	49.3
	> 50 years	76	50.7
Type of Stroke	Ischemic Stroke	106	70.7
	Hemorrhagic Stroke	44	29.3
Area of Stroke	Right Hemisphere	72	48.0
	Left Hemisphere	64	42.7
	Both	7	4.6
	Other*	7	4.6
Aphasia	Yes	22	14.7
	No	128	85.3
Recurrence	Yes	14	9.3
	No	136	90.7
NIHSS Score (N = 97)	Mild	21	21.6
	Moderate	25	25.8
	Severe	51	52.6
MMSE (Adjusted for education)	With Cognitive Impairment	25	16.7
	No Cognitive Impairment	125	83.3
Depression Status	Depression	65	43.3
	No Depression	85	56.7
Hypertension	Yes	105	70.0
	No	45	30.0
Diabetes	Yes	30	20.0
	No	120	80.0
Cardiac Disease	Yes	43	28.7
	No	107	71.3
HIV	Yes	11	7.3
	No	139	92.7

HIV (Human immunodeficiency virus), MMSE (Mini-mental State Exam), NIHSS (National Institute of Health Stroke Scale), PHQ-9 (Patient Health Questionnaire-9)

* Includes 3 (2%) patients with cerebellar area stroke and 2(1.3%) patients each for the subarachnoid area and venous area strokes. Additional comorbidities include chronic lower back pain (CLBP) in 11 (7.3%) patients, cancer in 5 (3.3%) patients, and peripheral neuropathy in 7 (4.7%) patients

### Disability and limitations in activities of daily living

**Fig. 1:**
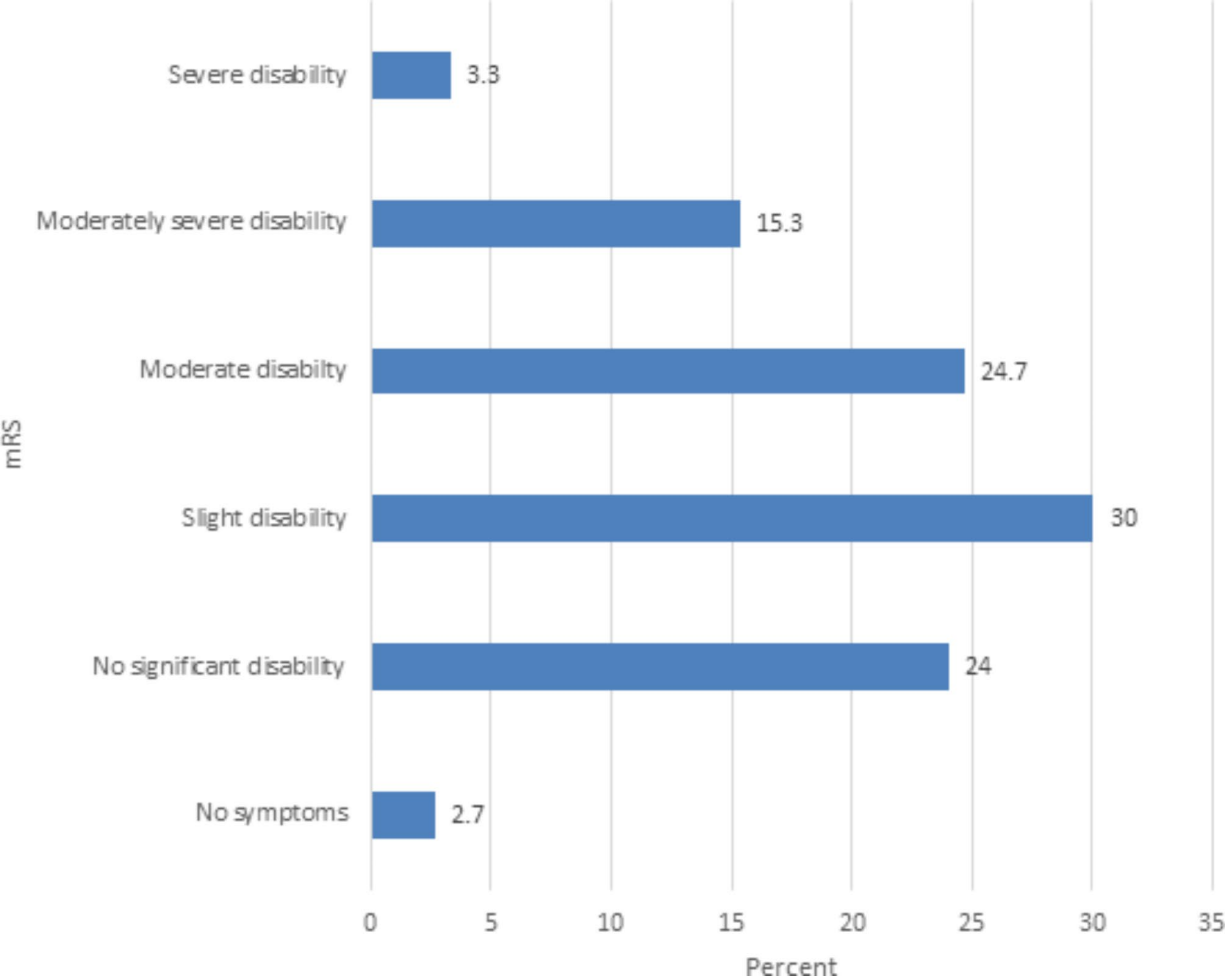
Modified Rankin Scale (mRS) scores of Stroke Patients at TASH, Neurology Clinic, April-October 2022

Respondents’ disability measured by the modified Rankin Scale (mRS) revealed moderate to severe disability (mRS of 3 to 5) among 65 (43.3%) of the participants which is denoted in Fig. 1. The level and severity of limitations among stroke survivors based on the BADL and IADL scale measurements are denoted in Table 3.

The median (IQR) BI score was 100 (84–100). The proportion of patients with limitations in basic ADL (dependent or had BI < 95) was 38%, while 79.3% of them had limitations in instrumental ADL (FAI ≤ 30). The median (IQR) FAI score was 16 [6–21, 25–29]. Regarding severity, 16.7% of stroke survivors were severely restricted from basic ADL (BI ≤ 60), while 50% of the study participants were severely restricted from instrumental ADL (FAI ≤ 15).

**Table 3:** Level and Severity of Limitations in BADL & IADL of Stroke Patients at TASH, Apr-Oct, 2022

VariablesCategories		Number%	
Limitations in BADL (BI < 95)	With Limitations No Limitations	57 93	38.0% 62.0%
Severe Limitations in BADL (BI ≤ 60)	Yes	25	16.7%
	No	125	83.3%
Limitations in IADL (FAI ≤ 30)	With Limitations	119	79.3%
	No Limitations	31	20.7%
Severe Limitations in IADL (FAI ≤ 15)	Yes	75	50.0%
	No	75	50.0%

ADL (Activities of Daily Living), BADL (Basic Activities of Daily Living), BI (Barthel Index), FAI (Frenchay Activity Index), and IADL (Instrumental Activities of Daily Living)

### Factors associated with limitations in activities of daily living among stroke patients included in the study

The factors crudely associated with post-stroke limitations in ADL were assessed using binary logistic regression models. To select the candidate variables of clinical importance, all variables with p < 0.25 were further analyzed in the final multivariable model. Accordingly, regular substance use (AOR = 11.1; 95% CI = 1.1-115.5) and cardiac disease as comorbidity (AOR = 7. 9; 5% CI = 1.3–37.5) were associated with limitations in BADL (as measured by the BI (< 95); while initial stroke severity (NIHSS ≥ 5) (AOR = 7.3; 95% CI = 1.2–44.7) was associated with limitations in IADL (FAI score of < 30).

In the same manner, the factors associated with severe activity limitations were also analyzed (BI ≤ 60 for BADL and FAI ≤ 15 for IADL). An increase in age was associated with severe limitations in BADL (AOR = 1.1; 95% CI = 1.04–1.15), while depression (PHQ9 score of ≥ 10) (AOR = 5.1; 95% CI = 1.1–23.2) was associated with severe restrictions in IADLs (Table 4).

**Table 4:** Factors associated with Post-stroke Limitations in Basic and Instrumental Activities of Daily Living in TASH, Apr-Oct, 2022

	% with Limitations in BADL (BI < 95);	% with Severe Limitations in BADL (BI ≤ 60);	% with Limitations in IADL (FAI ≤ 30);	%with Severe Limitations in IADL (FAI ≤ 15);
Characteristics	n(%); AOR (95% CI)	n(%); AOR (95% CI)	n(%); AOR (95% CI)	n(%); AOR (95% CI)
Age, Mean (SD)	57 (± 15);	**65(± 14);**	54(± 15);	57(± 16);
	0.985 (0.88,1.1)	1.1 (1.04–1.15)*	0.94(0.85,1.03)	0.99(0.9,1.1)
Duration Since	17 (31.5);	20 (20.8);	81 (84.4);	54(56.3);
Stroke ≤ One Year	5.18 (0.38,70.5)	0.000(0.000)	5.7 (0.007,4834)	0.84 (0.01,79.7)
(N = 96)				
History of Regular	**16 (64);**	4(16);	22(88);	16(64);
substance use	11.1 (1.1,115)*	0.000(0.000)	31(1.3,745)*	3.5 (0.3,38.1)
present (N = 25)^				
Age at Onset > 50	35 (46.1);	20(26.3);	67 (88.2);	45(59.2);
years (N = 76)	5.667 (0.77,45.2)	0.000(0.000)	0.249 (0.045,1.4)	0.29(0.068,1.25)
Comorbid Cardiac	**11 (25.6);**	7 (16.3);	38 (88.4);	24(55.8);
Disease (N = 43)	6.99 (1.3,37.5)*	2.1 (0.54,8.02	1.9 (0.17,20.1)	1.9(0.36,10.1)
Aphasia (N = 22)	13 (59.1);	8(36.4);	22 (100);	19(86.4);
	3.15 (0.33,29.9)	0.000(0.000)	0.000(0.000)	0.52(0.05,5.24)
Depression	43 (66.2);	22(33.8);	63 (96);	**58(89.2);**
(N = 65)	0.58 (0.06,5.3)	0.000(0.000)	0.167 (0.01,2.85)	5.1(1.1,23.2)*
Cognitive	19 (76);	15 (60);	25 (100);	23(92);
Impairment	0.21 (0.15,3.1)	0.000(0.000)	0.000(0.000)	5.8(0.4,83.3
(MMSE ≤ 23) (N = 25)				
Initial Stroke	32 (42.1);	16 (21.1);	**63 (82.9);**	47(61.8);
Severity (NIHSS	0.68 (0.04,10.86)	0.000(0.000)	7.33 (1.2,44.7)*	0.26(0.05,1.43)
≥ 5) (N = 76)				

BADL = Basic Activities of Daily Living; BI = Barthel Index; FAI = Frenchay Activity Index; IADL = Instrumental Activities of Daily Living; MMSE = Minimental State Examination; mRS = Modified Rankin’s Scale; NIHSS = National Institute of Health Stroke Scale; PHQ-9 = Patient Health Questionnaire-9. * represent statistically significant variables with p-values ≤ 0.05. ^Substances of abuse identified in our study include alcohol, nicotine, and khat (Catha edulis). N (capital) represents the number of participants with the mentioned characteristics, while “n”(small) represents the proportion of patients with limitations in ADL.

## Discussion

In this study, we sought to identify the level of limitation in basic and instrumental activities of daily living (BADL and IADL respectively) of stroke survivors using the Barthel and Frenchay Activity Indices, respectively. In addition, we further, assessed the contributory factors associated with basic and instrumental ADL.

Overall, the study identified a significant proportion of stroke survivors with activity limitation, in terms of both basic and instrumental ADL (38% and 79.3% respectively) with 16.7% and 50% of them having severe limitations in BADL and IADL, respectively. The data suggests that most of the study participants had some form of disability (73.3%) ranging from slight (mRS of 2) in 30%, to moderate to severe disability (mRS of 3–5) in 43.3% of the participants (Fig. 1). Our findings of post-stroke limitations in BADL are concordant with a prospective Israeli study, which documented a significant rate of limitations in BADL (42.3% of stroke survivors (BI < 95)). This same study revealed the proportion of limitations in IADL (restriction in participation, FAI < 30) to be 28.2% while our finding was 79.3%. The higher proportion of limitations in IADL observed in our study would be related to the number of stroke patients with a stroke duration of less than one year. Lower FAI scores at less than one-year post-stroke were seen in a population-based study, which revealed that 17% of the participants had the lowest possible score of 0 and the average FAI score was 17.4 [29].

Findings similar to our study were also noted in a prospective analysis of the South London Stroke Register which identified between 20% and 30% of stroke survivors to have a poor range of outcomes up to 10 years after stroke [30]. Another study conducted in the Western Cape, South Africa identified moderate to severe limitations in basic ADL (as assessed by the Barthel Index) among 30% of patients after 6 months of stroke onset [31].

Our study identified depression in 43.3% of the study participants. A local study conducted in two centers situated in Addis Ababa, Ethiopia also found a high prevalence of post-stroke depression from a total of 84 ischemic stroke patients (32.2%) [8]. Furthermore, in our study, hypertension was identified as the most common comorbidity (70%), while cardiac comorbidity was identified in 28.7% of the patients. In another study, which assessed the incidence and pattern of stroke among patients admitted to a ward at Yirgalem General Hospital, Southern Ethiopia, similar patterns of comorbidity distribution were identified, whereby hypertension accounted for 71% and cardiac disease (in the form of ischemic or valvular heart disease) accounted for 27.4% [19].

History of regular substance use was reported by 16.6% of participants in our study, which is low when compared with a population-based study in the US which assessed trends in substance abuse preceding stroke among young adults and documented 45–62% of them had substance abuse [20]. The higher numbers seen in the US study could be related to the cohort included in the study which mainly enrolled younger males (56%). Other likely attributes in this regard could be social desirability bias and self-report of substance use, with lower rates of self-report of substance use and underestimation being attested in a study conducted among urban substance users in Baltimore, USA [21]. Nonetheless, the observed findings cannot be overlooked since a significant proportion of regular substance users had limitations in basic (64%) and instrumental (88%) ADLs.

Our study identified regular substance to be associated with BADL, as patients with regular use had higher odds of activity limitations (p-value 0.044). Cardiac disease as a comorbidity was an independent predictor of BADL (p-value 0.023), while initial stroke severity (NIHSS) was a predictor of instrumental ADL (p-value 0.031). Furthermore, an increase in age was found to be associated with severe limitations in BADL (p-value < 0.001), while depression was found to be associated with severe restrictions in IADL (p-value 0.037). In the prospective Israeli study, age at stroke onset and degree of disability were identified as predictors [14], while an Italian study identified impaired mood (depression) as independent predictors of FAI indoor and outdoor activities respectively [22]. In our study, older age at stroke onset was associated with a higher degree of activity limitation, but statistical significance was not met.

Nonetheless, this study was not without its key findings; unlike the studies mentioned above, our study identified cardiac comorbidity and regular substance use as predictors of limitations in BADL. A Serbian study that assessed the impact of comorbidity on rehabilitation outcomes after ischemic stroke identified cardiac illnesses (atrial fibrillation, myocardial infarction, and dilated cardiomyopathy) to be associated with a dismal outcome [23].

In addition, in keeping with prior studies, predictor variables such as employment, type of stroke, location of the stroke, duration since the stroke, stroke recurrence, aphasia, and cognitive impairment were assessed for possible association with ADL, but statistical significance was not met. Employment, type, and location of stroke were not significantly associated with limitations in ADL in the studies conducted in Israel and Italy [14, 22]. Further studies with emphasis to the type of stroke and activity limitation are warranted as the presentation, pathophysiology, and prognosis of small vessel ischemic changes are different from other stroke subtypes [32]. There is a paucity of data when it comes to recurrent stroke and limitations in ADL, as most of the available studies assess only patients with first-ever strokes. In our study, there were merely 14(9.3%) patients with stroke recurrence; hence, a large-scale study is needed to further assess patients with recurrent strokes. Previous studies show that cognitive impairment after a stroke is common and leads to post-stroke dementia. Post-stroke cognitive impairment prevalence varies from 23 to 55% three months after stroke, ending with a decline (11–31%) one year after stroke onset [24, 33, 34]. In this study, 16.7% of the patients had cognitive impairment; although higher rates of activity limitations were seen in patients with cognitive impairment (76% for BADL and 100% for IADL), the findings were not statistically significant. Aphasia was found to be associated with limitations in ADL in a French study that assessed a cohort of 36 aphasic patients [35]. In our study, aphasia was identified in 14.7% of them; higher rates of activity limitation (59.1% for BADL and 100% for IADL) were seen with aphasic patients though not statistically significant. The relatively lower magnitude of aphasia and cognitive impairment, as opposed to the French study in our patient cohort, could contribute to this finding; warranting further large-scale studies.

### Strengths and limitations of the study

This study is the first of its kind using a standardized tool for measuring post-stroke limitations in activities of daily living in our setting. In addition, this novel study had a 100% response rate. It further elucidated key factors that need subsequent studies. Nonetheless, the study was not without its limitations. The small sample size used makes it difficult to generalize for the general population. Due to our limited sample size, some variables were clumped together. Hence, dichotomizing variables such as regular substance use may have disproportionately exaggerated our findings. In addition, the use of a non-probability sampling technique might have introduced some bias. Another key source of bias in this hospital-based study is the exclusion of stroke survivors who were unable to attend our stroke clinic, resulting in failure to capture activity limitations in either spectrum of the disease (severely debilitated patients who have defaulted from further follow-up in a tertiary healthcare setting or patients with no significant disability who have been discharged to a primary healthcare setting). Hence, since it is a sample from a tertiary hospital, it may not represent the range of stroke severity at a national level. As with any other cross-sectional study design, the “egg and chicken” dilemma holds true in establishing an etiologic association among variables.

### Conclusion and recommendations

In conclusion, limitations in activities of daily living after stroke is a common phenomenon in our setting as seen in other parts of the world. Identification and directed rehabilitative measures for limitations in ADL may enhance patient recovery. Our study demonstrated age, degree of disability, initial stroke severity, regular substance use, cardiac comorbidity and depression to be associated with limitations in ADL. Hence, screening all post-stroke patients to identify both basic and instrumental ADL is essential. Developing a framework capturing ADL as a stroke outcome is key for the proper management of stroke patients.

### Electronic supplementary material

Below is the link to the electronic supplementary material.

Supplementary Material 1

## Data Availability

All data generated or analyzed are included in this published article.

## Abbreviations

AAU: Addis Ababa University
ADL: Activities of Daily Living
AOR: Adjusted Odd’s Ratio
ASA: American Stroke Association
BADL: Basic Activities of Daily Living
BI: Barthel Index
COR: Crude Odd’s Ratio
FAI: Frenchay Activity Index
FMOH: Federal Ministry of Health
IADL: Instrumental Activities of Daily Living
IBM: International Business Machine
ICF: International Classification of Functioning
IQR: Interquartile Range
HIV: Human Immunodeficiency Virus
HRQOL: Health-Related Quality of Life
LMIC: Low and Middle-Income Countries
MMSE: Mini-mental Status Exam
mRS: modified Rankin Scale
NIHSS: National Institute of Health Stroke Scale
OR: Odd’s Ratio
PHQ: 9-Patient Health Questionnaire-9
SPSS: Statistical Package for Social Sciences
TASH: Tikur Anbessa Specialized Hospital
UNCRPD: The United Nations Convention on the Rights of Persons with Disabilities
USA: United States of America
WHO: World Health Organization
WSO: World Stroke Organization
