# Supplementary material for: Post-stroke limitations in daily activities: experience from a tertiary care hospital in Ethiopia
Source: BMC Neurol. 2023 Oct 9;23:364. doi: 10.1186/s12883-023-03419-9 (PMC10561502; doi:10.1186/s12883-023-03419-9)
Supplement: Supplementary file 1 — Supplementary Material 1 [file 12883_2023_3419_MOESM1_ESM.docx]

Table S1. Factors associated with Poststroke Limitations in Basic Activities of Daily Living, among Stroke patients in TASH, April-October, 2022.

|  | | **Basic ADL (assessed by BI)** | | | | |
| --- | --- | --- | --- | --- | --- | --- |
|  |  | No Limitations | Has Limitations |  |  |  |
|  |  |  |  | COR | AOR | P-Value |
| **Age (Mean (SD))** | | 50(14) | 57 (15) | 1.03 (1.008-1.058) | 0.985 (0.88-1.1) | 0.793 |
| **Regular Substance Use? (%)** | No | 67.2 | 32.8 |  |  |  |
|  | **Yes** | **36.0** | **64.0** | **3.642 (1.484,6.072** | **11.1 (1.1,115.5)** | **0.044** |
| **Cardiac Disease (%)** | No | 57.0 | 43.0 |  |  |  |
|  | **Yes** | **74.4** | **25.6** | **2.2 (1.001,4.8)** | **6.99 (1.3,37.5)** | **0.023** |
| **Aphasia (%)** | No | 65.6 | 34.4 |  |  |  |
|  | Yes | 40.9 | 59.1 | 2.76 (1.1-6.9) | 3.15 (0.33-29.9) | 0.317 |
| **Cognitive Impairment (MMSE) (%)** | No | 69.6 | 30.4 |  |  |  |
|  | Yes | 24.0 | 76.0 | 7.25 (2.7-19.6) | 0.21 (0.15-3.1) | 0.254 |
| **Depression (PHQ-9) (%)** | No | 83.5 | 16.5 |  |  |  |
|  | Yes | 33.8 | 66.2 | 9.9 (4.6-21.4) | 0.58 (0.06-5.3) | 0.63 |
| **Initial Stroke Severity (NIHSS above 5) (%)** | No | 85.7 | 14.3 |  |  |  |
|  | Yes | 57.9 | 42.1 | 4.36 (1.2-16) | 0.68 (0.04-10.86) | 0.783 |

*AOR (Adjusted Odds Ratio), BADL (Basic Activities of Daily of Living), BI (Barthel Index), COR (Crude Odds Ratio), IQR(Interquartile range), MMSE (Minimental State Examination), mRS (Modified Rankin’s Scale), NIHSS (National Institute of Health Stroke Scale), PHQ-9 (Patient Health Questionnaire-9), SD (Standard deviation). Statistically significant findings are represented in bold.*

Table S2. Factors associated with Poststroke Limitations in Instrumental Activities of Daily Living, among Stroke patients in TASH, April-October, 2022.

|  | | **Instrumental ADL (assessed by FAI)** | | |  |  |
| --- | --- | --- | --- | --- | --- | --- |
|  |  | No Limitations | Has Limitations |  |  |  |
|  |  |  |  | COR | AOR | P-value |
| **Age, Mean (SD)** | | 47 (11) | 54(15) | 1.03 (1.01-1.1) | 0.17 (0.01-2.9) | 0.217 |
| **Regular Substance Use? (%)** | No | 22.4 | 77.6 |  |  |  |
|  | **Yes** | **12.0** | **88.0** | **2.12 (0.6-7.6)** | **31 (1.31,745)** | **0.034** |
| **Cardiac Disease (%)** | No | 24.3 | 75.7 |  |  |  |
|  | Yes | 11.6 | 88.4 | 2.44 (0.87-6.8) | 1.86 (0.17-20.4) | 0.613 |
| **Depression (PHQ-9) (%)** | No | 34.1 | 65.9 |  |  |  |
|  | Yes | 3.1 | 96.9 | 16.3 (3.72-71.5) | 0.17 (0.01-2.85) | 0.217 |
| **Initial Stroke Severity (NIHSS ≥5) (%)** | No | 19.0 | 81.0 |  |  |  |
|  | **Yes** | **17.1** | **82.9** | **0.88 (0.25-3.04)** | **7.33 (1.2,44.7)** | **0.031** |

*AOR (Adjusted Odds Ratio), COR (Crude Odds Ratio), FAI (Frenchay Activity Index), IADL (Instrumental Activities of Daily Living), IQR (Interquartile Range), MMSE (Minimental State Examination), mRS (Modified Rankin’s Scale), NIHSS (National Institute of Health Stroke Scale), PHQ-9 (Patient Health Questionnaire-9), SD (Standard deviation). Statistically significant findings are represented in bold.*

Table S3. Factors associated with severe limitations in Basic Activities of Daily Living, among Stroke patients in TASH, April-October, 2022.

|  | | **Severe Limitation in BADL (BI≤60)** | | | | |
| --- | --- | --- | --- | --- | --- | --- |
|  |  | No | Yes |  |  |  |
|  |  | N (%) | N (%) | COR | AOR | P-value |
| **Age, Mean (SD)** | | **51(14)** | **65(14)** | **1.08 (1.04-1.13)** | **1.1 (1.04-1.15)** | **<0.001** |
| **Age at Onset** | ≤ 50 years | 69 (93.2) | 5 (6.8) |  |  |  |
|  | > 50 years | 56 (73.7) | 20 (26.3) | 4.9 (1.74-14) | 0.001 | 0.998 |
| **Aphasia** | No | 111 (86.7) | 17 (13.3) |  |  |  |
|  | Yes | 14 (63.6) | 8 (36.4) | 3.7 (1.4-10.2 | 0.000-0.001 | 0.993 |
| **Depression (PHQ9≥10)** | No | 82 (96.5) | 3 (3.5) |  |  |  |
|  | Yes | 43 (66.2) | 22 (33.8) |  | 0.00(0.00) | 0.994 |

*AOR (Adjusted Odds Ratio), BADL (Basic Activities of Daily of Living), BI (Barthel Index), COR (Crude Odds Ratio), IQR(Interquartile Range), mRS (Modified Rankin’s Scale), PHQ-9 (Patient Health Questionnaire-9), SD (Standard deviation). Statistically significant findings are represented in bold.*

Table S4. Factors associated with severe limitations in Instrumental Activities of Daily Living, among Stroke patients in TASH, April-October, 2022.

|  | | **Severe Limitation in IADL (FAI ≤15)** | | | | |
| --- | --- | --- | --- | --- | --- | --- |
|  |  | No | Yes |  |  |  |
|  |  | N (%) | N (%) | COR | AOR | P-value |
| **Age, Mean (SD)** | | 49 (12) | 57 (16) | 1.04 (1.02-1.07) | 1.02 (0.92-1.14) | 0.705 |
| **Age at Onset** | ≤ 50 years | 44 (59.5) | 30 (40.5) |  |  |  |
|  | > 50 years | 31 (40.8) | 45 (59.2) | 2.13 (1.11-4.09) | 0.3 (0.7-1.25) | 0.096 |
| **Aphasia** | No | 72 (56.3) | 56 (43.8) |  |  |  |
|  | Yes | 3 (13.6) | 19 (86.4) | 8.14 (2.3-29) | 0.57 (0.06-4.9) | 0.576 |
| **Depression (PHQ9≥10)** | No | 68 (80) | 17 (20) |  |  |  |
|  | **Yes** | **7 (10.8)** | **58 (89.2)** | **33.1 (12.9-85.4)** | **5.1 (1.1-23.2)** | **0.037** |

*AOR (Adjusted Odds Ratio), COR (Crude Odds Ratio), FAI (Frenchay Activity Index), IADL (Instrumental Activities of Daily Living), IQR (Interquartile Range), mRS (Modified Rankin’s Scale), PHQ-9 (Patient Health Questionnaire-9), SD (Standard deviation). Statistically significant findings are represented in bold.*
